# Fast and Robust Optical Cooling via Shortcut to Adiabaticity

**DOI:** 10.3390/e27080851

**Published:** 2025-08-11

**Authors:** Zhiyu Wang, Jie Lu

**Affiliations:** 1 Department of Physics, Shanghai University, Shanghai 200444, China; wangzhiyu@shu.edu.cn; 2 Institute for Quantum Science and Technology, Shanghai University, Shanghai 200444, China

**Keywords:** shortcuts to adiabaticity, optical cooling, robust quantum control

## Abstract

Optical cooling is a key technique for preparing ultracold atoms in quantum technologies and precision experiments. We employ shortcut-to-adiabaticity (STA) techniques to accelerate and stabilize laser-based atomic cooling protocols. This approach improves the performance of conventional adiabatic momentum transfer schemes by addressing key limitations such as Doppler shifts, laser intensity fluctuations, and spontaneous emission. We first examine two- and three-level atomic systems subjected to counter-propagating laser pulses that induce momentum reduction through photon recoil. STA methods are then employed to construct pulse sequences that are robust against detuning errors and amplitude noise, outperforming standard π-pulse schemes in resilience. Meanwhile, we analyze the dissipative dynamics during the momentum transfer and demonstrate the superiority of the STA protocol in enhancing momentum transfer efficiency via accelerated control. The results demonstrate that STA can significantly improve both the efficiency and robustness of cooling. These findings have implications for applications in atomic physics, quantum information processing, and precision metrology.

## 1. Introduction

Since the 1970s, light–matter interactions have been instrumental in controlling atomic motion, with radiation pressure and dipole forces enabling precise manipulation of quantum states [1,2,3,4,5]. By carefully tailoring laser parameters, researchers have been able to cool, accelerate, or confine atoms, capabilities that underpin groundbreaking technologies such as optical lattice clocks [6,7], matter-wave interferometers [8], and Bose–Einstein condensates [9,10,11]. In laser cooling, velocity-selective optical forces counteract thermal motion via photon recoil, reaching sub-Doppler temperatures essential for quantum degeneracy [12,13]. These advancements have laid the foundation for a broad spectrum of applications, from quantum simulation and metrology to quantum information processing and the precision control of atomic and molecular systems [14,15,16,17].

While laser cooling techniques exploit resonant optical transitions to impart momentum impulses, their practical implementation faces several limitations that affect performance and scalability. Multi-level atomic structures introduce spontaneous emission into dark states, leading to population trapping and stochastic recoil, which broadens the momentum distribution. Additionally, Doppler shifts complicate velocity-selective cooling, as faster atoms require dynamically detuned resonant lasers to maintain efficiency. Fluctuations in laser intensity further degrade coherence, inducing diabatic transitions and reducing the fidelity of momentum transfer. To address these issues, strategies such as bichromatic fields [18,19,20] and chirped laser pulses [21,22,23,24] have been proposed to enhance stimulated transitions and suppress spontaneous decay. While these approaches improve cooling efficiency, they are often sensitive to noise and control imperfections, especially in complex multi-level systems. As a result, a universal and robust laser cooling strategy remains elusive, particularly for high-dimensional quantum systems and dynamic experimental conditions.

**Figure 1:**
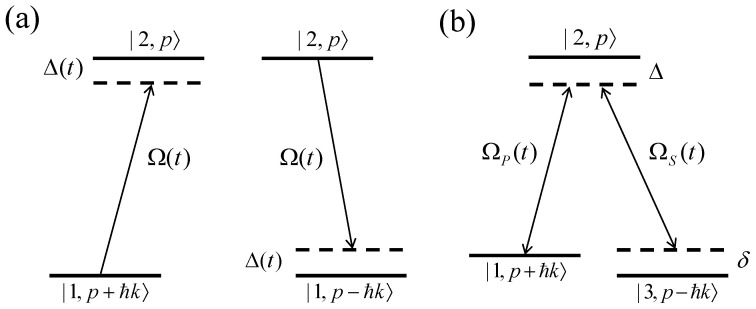
Schematic diagrams of optical cooling based on adiabatic passage. (**a**) Two-level scheme with counter-propagating pulses characterized by a time-dependent Rabi frequency Ω(t) and detuning Δ(t), implementing a closed-loop momentum transfer via adiabatic rapid passage (RAP) transitions: |1,p+ℏk〉→|2,p〉→|1,p−ℏk〉. (**b**) Three-level scheme using pump $\Omega_{P}\left(t\right)$ and Stokes $\Omega_{S}\left(t\right)$ pulses to implement a stimulated Raman adiabatic passage (STIRAP) protocol between motional states, coherently transferring the population from |1,p+ℏk〉 to |3,p−ℏk〉 through a dark state, minimizing spontaneous emission.

Traditional techniques like adiabatic rapid passage (RAP) and stimulated Raman adiabatic passage (STIRAP) have been used to drive coherent momentum exchanges between atoms and light (see Figure 1), yielding strong optical forces. These methods, while robust to parameter variations, require long operation times that make them vulnerable to environmental decoherence [25,26,27,28]. Recently, shortcuts to adiabaticity (STA) have emerged as a powerful approach that accelerates these processes while maintaining error resilience [29,30,31]. STA protocols, based on counterdiabatic driving or invariant-based inverse engineering [32], allow for rapid state transformations without sacrificing robustness. The integration of invariant-based inverse engineering with optimal control theory further refines STA protocols, optimizing robustness against systematic errors and decoherence and exceeding conventional adiabatic limits [33,34,35,36]. A notable development in this area was presented in Ref. [37], where inverse engineering based on Lewis–Riesenfeld invariants was successfully applied to achieve rapid momentum transfer using counter-propagating red– and blue–detuned pulses. Although this approach mitigates some issues, such as sensitivity to Doppler shifts, intensity fluctuations, and spontaneous emission, it does not fully optimize the process. In particular, the fixed protocol structure limits adaptability to unforeseen disturbances or parameter drifts.

In this article, we present a framework that leverages shortcuts to adiabaticity (STA) to realize fast and robust optical cooling, enhancing conventional RAP and STIRAP protocols. We begin with a two-level optical cooling scheme, illustrated in Figure 1a, where the momentum transfer mechanism is driven by single-photon recoil in an RAP cycle. In this setup, a pair of counter-propagating laser pulses induce internal transitions between two states, each associated with distinct momentum sidebands. By employing red- and blue-detuned pulses, a closed-loop momentum transfer cycle is realized, resulting in a net change in atomic momentum. Building on this, we extend the approach to a three-level optical cooling scheme based on STIRAP, as shown in Figure 1b. This scheme operates under two-photon resonance with large, fixed single-photon detuning. Counter-propagating pump and Stokes pulses are applied in a counter-intuitive sequence, with the Stokes pulse preceding the pump. This pulse ordering drives an adiabatic population transfer between momentum states through a dark state while suppressing occupation of the excited state. As a result, the system undergoes a net momentum transfer with minimal spontaneous emission, making it highly effective for sub-recoil cooling. To further enhance the robustness and efficiency of both RAP- and STIRAP-based cooling schemes, we employ STA techniques to design laser pulse shapes that are resilient against Doppler shifts and pulse-shaping imperfections. These STA protocols ensure optimal control strategies even under realistic experimental constraints, such as finite laser intensity and limited momentum resolution.

This paper is organized as follows: Section 2 introduces the preliminaries for the model and the Hamiltonian for optical cooling. Section 3 presents the detailed results obtained from optimal STA protocols with respect to systematic errors induced by Doppler shifts or laser intensity fluctuations. Finally, Section 4 provides the summary and outlook.

## 2. Preliminaries: Model and Hamiltonian

### 2.1. RAP Cooling

We consider the laser cooling process in a two-level atomic system [38,39], where the atom interacts with a classical monochromatic electric field described by

(1) $$E\left(x,t\right)=\frac{1}{2}\hat{\epsilon }\;E_{0}\left(e^{\pm ikx+i\omega_{i}t}+c.c.\right),$$

with polarization vector $\hat{\epsilon }$, field amplitude $E_{0}$, laser frequency $\omega_{i}$, and wave vector *k*. The spatial dependence of the field induces quantized momentum transitions, represented by the identity

(2) $$e^{\pm ikx}=\sum_{p}|p\rangle \langle p\mp \hbar k|,$$

which describes how photon absorption and stimulated emission impart momentum kicks of ±ℏk to the atom [13]. These discrete momentum exchanges underpin laser cooling: atoms undergoing repeated absorption–emission cycles receive net momentum kicks opposing their motion, leading to a gradual reduction in kinetic energy.

The system Hamiltonian, encompassing both internal and external degrees of freedom, is given by

(3) $$H=\frac{p^{2}}{2m}+\frac{\hbar }{2}\omega_{12}\sigma_{z}+\frac{\hbar }{2}\Omega \left(\sigma e^{\pm ikx+i\omega_{i}t}+H.c.\right),$$

where the positive sign in $e^{\pm ikx}$ corresponds to excitation (Figure 1a) and the negative sign corresponds to stimulated emission. Here, $\sigma_{z}=\left|2\rangle \langle 2\right|-\left|1\rangle \langle 1\right|$ is the Pauli-*z* operator, σ=|1〉〈2| is the lowering operator, Ω denotes the Rabi frequency characterizing the atom–field interaction strength, and $\omega_{12}$ is the transition frequency between the two internal atomic states.

To facilitate the application of the STA protocol, we apply the unitary transformation defined by the operator

(4) $$U\left(t\right)=e^{-i\phi_{1}\left(t\right)}e^{\pm ikx}\left|1\rangle \langle 1\right|+e^{-i\phi_{2}\left(t\right)}|2\rangle \langle 2|,$$

where the time-dependent phases $\phi_{1}\left(t\right)$ and $\phi_{2}\left(t\right)$ are chosen as [40]

(5) $$\phi_{1}\left(t\right)=\left[\frac{{\left(p\pm \hbar k\right)}^{2}}{2m\hbar }-\frac{\omega_{12}}{2}+\frac{\omega_{i}}{2}\right]t,\;\phi_{2}\left(t\right)=\left[\frac{p^{2}}{2m\hbar }+\frac{\omega_{12}}{2}-\frac{\omega_{i}}{2}\right]t.$$

This transformation U(t) defines a new basis, the dressed states:

(6) $$|\tilde{1}\rangle =e^{-i\phi_{1}\left(t\right)}\left|1,p\pm \hbar k\rangle ,\;\right|\tilde{2}\rangle =e^{-i\phi_{2}\left(t\right)}|2,p\rangle .$$

The Hamiltonian in the original frame is transformed according to $\tilde{H}=U^{†}HU-i\hbar U^{†}\partial_{t}U$. Substituting U(t) and carrying out the calculation yields the simplified form in the dressed basis:

(7) $$\tilde{H}=\frac{\hbar }{2}\left(\Delta {\tilde{\sigma }}_{z}+\Omega {\tilde{\sigma }}_{x}\right),$$

where ${\tilde{\sigma }}_{z}=|\tilde{2}\rangle \langle \tilde{2}\left|-\right|\tilde{1}\rangle \langle \tilde{1}|$ and ${\tilde{\sigma }}_{x}=|\tilde{2}\rangle \langle \tilde{1}\left|+\right|\tilde{1}\rangle \langle \tilde{2}|$.

The corrected detuning Δ is defined as

(8) $$\Delta =\omega_{i}-\omega_{12}\mp \frac{kp}{m}-\frac{\hbar k^{2}}{2m},$$

with the resonance condition Δ=0 when

(9) $$\omega_{i}=\omega_{12}\pm \frac{kp}{m}+\frac{\hbar k^{2}}{2m}.$$

Here, the term ±kp/m arises from the Doppler effect due to atomic motion, while $\hbar k^{2}/2m$ corresponds to the photon recoil energy [41]. For a thermal atomic momentum distribution $P\left(p\right)\propto \exp\left[-{\left(p-p_{0}\right)}^{2}/2\sigma_{p}^{2}\right]$, atoms with momentum offset from $p_{0}$ experience detuning errors, $\delta_{\Delta }=k\left(p-p_{0}\right)/m$, even when tuned to resonance at $p_{0}$. This momentum-dependent detuning limits cooling efficiency since faster atoms with larger $|p-p_{0}|$ experience stronger deceleration forces.

In addition, laser intensity fluctuations cause variations in the Rabi frequency, modeled as $\Omega \to \Omega (1+\delta_{\Omega })$, where $\delta_{\Omega }$ quantifies the relative intensity noise, arising mainly from quantum shot noise in photon counting [42]. These fluctuations translate into stochastic errors in the atom–field coupling strength. Together, the detuning errors $\delta_{\Delta }$ and intensity noise $\delta_{\Omega }$ define fundamental precision limits for momentum-selective cooling protocols.

Crucially, spontaneous emission from the finite excited-state lifetime induces stochastic momentum diffusion. Photons emitted randomly impart recoil momentum, redistributing atoms outside the target momentum subspace. This momentum leakage degrades coherence in momentum reduction and ultimately constrains cooling efficiency.

To mitigate these challenges, STA-enhanced RAP cooling offers significant advantages. By dramatically shortening operation times via optimized pulse shaping, spontaneous emission is effectively suppressed. Moreover, STA protocols exhibit inherent robustness against parameter fluctuations, notably Doppler-induced frequency shifts and laser intensity variations. This combination of accelerated dynamics and error-resilient control paves a promising path to overcoming the fundamental limitations of conventional two-level cooling schemes.

### 2.2. Stimulated Adiabatic Passage for Momentum Transfer

To enhance cooling efficiency beyond the two-level system previously discussed, we consider a three-level Λ-configuration with two ground states (|1〉, |3〉) and one excited state (|2〉). The driving optical field for Raman transitions is

(10) $$E\left(x,t\right)=\frac{1}{2}\left({\hat{\epsilon }}_{P}E_{P}e^{ikx+i\omega_{P}t}+{\hat{\epsilon }}_{S}E_{S}e^{-ikx+i\omega_{S}t}\right)+c.c.,$$

where counter-propagating lasers with frequencies $\omega_{P}$ and $\omega_{S}$ address moving atoms. Atoms moving opposite to the pump laser ($\omega_{P}$) experience Doppler-shifted resonance, absorbing photons and receiving a net momentum transfer of −2ℏk per transition cycle, leading to deceleration. The system Hamiltonian is [40]

(11) $$H=\frac{p^{2}}{2m}+\sum_{j=1}^{3}\hbar \omega_{j}|j\rangle \langle j|+\frac{\hbar }{2}\left(\Omega_{P}|2\rangle \langle 1|e^{ikx+i\omega_{P}t}+\Omega_{S}|2\rangle \langle 3|e^{-ikx+i\omega_{S}t}\right)+H.c.,$$

with Rabi frequencies $\Omega_{P}$ and $\Omega_{S}$ coupling transitions |1〉↔|2〉 and |3〉↔|2〉, respectively. For a counterintuitive adiabatic process, the pulse ratio must satisfy $\Omega_{P}/\Omega_{S}\to 0$ at t=0 and $\Omega_{P}/\Omega_{S}\to \infty$ at t=T.

Applying the unitary transformation

(12) $$U\left(t\right)=e^{-i\phi_{1}}e^{ikx}|1\rangle \langle 1|+e^{-i\phi_{2}}|2\rangle \langle 2|+e^{-i\phi_{3}}e^{-ikx}|3\rangle \langle 3|$$

yields the dressed basis

(13) $$\begin{array}{cc} |\tilde{1}\rangle & =e^{-i\phi_{1}\left(t\right)}|1,p+\hbar k\rangle ,\\ |\tilde{2}\rangle & =e^{-i\phi_{2}\left(t\right)}|2,p\rangle ,\\ |\tilde{3}\rangle & =e^{-i\phi_{3}\left(t\right)}|3,p-\hbar k\rangle , \end{array}$$

where

(14) $$\begin{array}{cc} \phi_{1}\left(t\right) & =[{\left(p+\hbar k\right)}^{2}/\left(2m\hbar \right)+\omega_{1}]t,\\ \phi_{2}\left(t\right) & =[{\left(p+\hbar k\right)}^{2}/\left(2m\hbar \right)+\omega_{2}-\Delta_{P}]t,\\ \phi_{3}\left(t\right) & =[{\left(p+\hbar k\right)}^{2}/\left(2m\hbar \right)+\omega_{3}-\Delta_{P}+\Delta_{S}]t, \end{array}$$

with detunings $\Delta_{P}=\omega_{P}-\omega_{2}+\omega_{1}$ and $\Delta_{S}=\omega_{S}-\omega_{2}+\omega_{3}$. The transformed Hamiltonian is

(15) $$\tilde{H}=\hbar \Delta |\tilde{2}\rangle \langle \tilde{2}|+\hbar \delta |\tilde{3}\rangle \langle \tilde{3}|+\frac{\hbar \Omega_{P}}{2}|\tilde{1}\rangle \langle \tilde{2}|+\frac{\hbar \Omega_{S}}{2}|\tilde{3}\rangle \langle \tilde{2}|+H.c.,$$

with single-photon detuning $\Delta =\Delta_{P}-kp/m-\hbar k^{2}/\left(2m\right)$ and two-photon detuning $\delta =\Delta_{P}-\Delta_{S}-2kp/m$.

The atomic wavefunction in this basis is $|\Psi (t)\rangle =c_{1}\left(t\right)|\tilde{1}\rangle +c_{2}\left(t\right)|\tilde{2}\rangle +c_{3}\left(t\right)|\tilde{3}\rangle$. To suppress spontaneous emission, we assume large detuning ($\left|\Delta \right|\gg \Omega_{P},\Omega_{S},p^{2}/\left(2m\hbar \right)$), enabling adiabatic elimination of $c_{2}\left(t\right)$:

(16) $$c_{2}\left(t\right)=\frac{\Omega_{P}}{2\Delta }c_{1}\left(t\right)+\frac{\Omega_{S}}{2\Delta }c_{3}\left(t\right).$$

This reduces the system to an effective two-level model between $|\tilde{1}\rangle$ and $|\tilde{3}\rangle$ with effective Rabi frequency $\Omega_{\mathrm{eff}}=\Omega_{P}\Omega_{S}/\left(2\Delta \right)$ and detuning $\Delta_{\mathrm{eff}}=\left(\Omega_{S}^{2}-\Omega_{P}^{2}\right)/\left(4\Delta \right)$ [43,44].

Momentum-dependent detuning errors arise from the two-photon resonance condition $\delta_{\Delta }=2k\left(p-p_{0}\right)/m$. Laser intensity fluctuations ($\Omega_{j}\to \Omega_{j}\left(1+\delta_{\Omega }\right)$) modify the effective parameters as $\Omega_{\mathrm{eff}}\to \Omega_{\mathrm{eff}}{\left(1+\delta_{\Omega }\right)}^{2}$ and $\Delta_{\mathrm{eff}}\to \Delta_{\mathrm{eff}}{\left(1+\delta_{\Omega }\right)}^{2}$, amplifying noise quadratically. The closed-loop transition cycle transfers a net momentum of −2ℏk per cycle while avoiding dark states and spontaneous emission, enabling optimized cooling through parameter control.

## 3. STA and Optimization

Within the closed-loop momentum transfer cycle, transitions between $|\tilde{1}\rangle$ and $|\tilde{2}\rangle$ exhibit symmetric dynamics governed by identical Rabi frequency Ω(t) and detuning Δ(t) profiles. Numerical simulations confirm that pulse sequences {Ω(t),Δ(t)} optimized for $|\tilde{1}\rangle \to |\tilde{2}\rangle$ yield identical fidelity when applied to the reverse transition $|\tilde{2}\rangle \to |\tilde{1}\rangle$. This symmetry originates from the form invariance of the two-level Schrödinger equation in the rotating frame, where both processes share the Hamiltonian (7). Consequently, we restrict subsequent pulse optimization and robustness analysis to the $|\tilde{1}\rangle \to |\tilde{2}\rangle$ transition, with solutions directly applicable to the reverse process.

### 3.1. Inverse Engineering Based on Dynamical Invariants

Using Lewis–Riesenfeld invariant theory, [45]

(17) $$\partial I/\partial t+\left(1/i\hbar \right)[I\left(t\right),\tilde{H}\left(t\right)]=0,$$

we construct the invariant [34]

(18) $$I\left(t\right)=\frac{\hbar \Omega_{0}}{2}\left[\cos\theta \left(t\right){\tilde{\sigma }}_{z}+\sin\theta \left(t\right)\cos\beta \left(t\right){\tilde{\sigma }}_{x}+\sin\theta \left(t\right)\sin\beta \left(t\right){\tilde{\sigma }}_{y}\right],$$

where $\Omega_{0}$ maintains dimensional consistency and ${\tilde{\sigma }}_{y}=-i\left(|\tilde{1}\rangle \langle \tilde{2}|-|\tilde{2}\rangle \langle \tilde{1}|\right)$. The control parameters are determined by

(19) $$\begin{array}{cc} \Omega & =-\dot{\theta }/\sin\beta ,\\ \Delta & =-\Omega \cot\theta \cos\beta -\dot{\beta }. \end{array}$$

The perturbed Hamiltonian ${\tilde{H}}^{\prime }$ incorporates two dominant error sources: (i) Doppler shifts $\Delta \to \Delta +\delta_{\Delta }$ modeled by ${\tilde{H}}^{\prime }=\left(\hbar /2\right)\delta_{\Delta }{\tilde{\sigma }}_{z}$ and (ii) laser intensity fluctuations $\Omega \to \Omega (1+\delta_{\Omega })$. Time-dependent perturbation theory [33] gives the inversion probability for Doppler errors:

(20) $$P\approx 1-{\left(\frac{\delta_{\Delta }}{2}\right)}^{2}{\left|\int_{0}^{T}\sin\theta e^{i\eta (t)}dt\right|}^{2},$$

where $\eta \left(t\right)=\int_{0}^{t}\left(\dot{\theta }\cot\beta /\sin\theta \right)dt^{\prime }$ is the Lewis–Riesenfeld phase. Using the expansion η(t)≈2θ+αsin(2θ) [35], with α denoting the first-order coefficient in the Fourier expansion of the Lewis–Riesenfeld phase η(t), and the polynomial ansatz $\theta \left(t\right)=\sum_{j=0}^{3}a_{j}t^{j}$ with boundary conditions

(21) $$\theta \left(0\right)=\pi ,\;\theta \left(T\right)=0,\;\dot{\theta }\left(0\right)=0,\;\dot{\theta }\left(T\right)=0,$$

we derive cotβ(t)=2sinθ[1+αcos(2θ)]. Setting α=−0.434 nullifies Doppler errors by enforcing $\left|\int_{0}^{T}\sin\theta e^{i\eta (t)}dt\right|=0$.

For intensity fluctuations $\Omega \to \Omega (1+\delta_{\Omega })$, the perturbation ${\tilde{H}}^{\prime }=\left(\hbar /2\right)\delta_{\Omega }\Omega {\tilde{\sigma }}_{x}$ yields

(22) $$P\approx 1-\delta_{\Omega }^{2}{\left|\int_{0}^{T}\dot{\theta }\sin^{2}\theta e^{i\eta (t)}dt\right|}^{2}.$$

**Figure 2:**
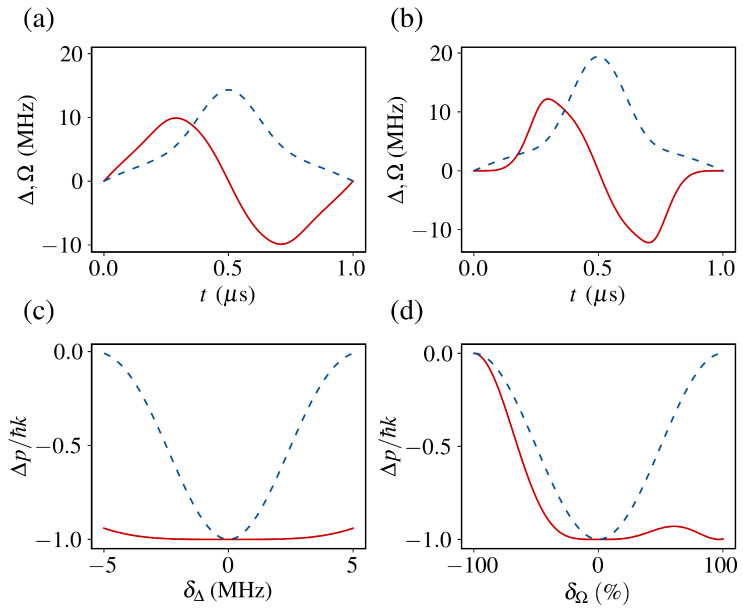
Pulse profiles and deceleration performance for the two-level system. (**a**,**b**) Temporal profiles of the designated detuning Δ (red solid line) and Rabi frequency Ω (blue dashed line) for α=−0.434 and α=−1. (**c**,**d**) Comparison of deceleration efficiency between the shortcuts to adiabaticity (STA)-optimized pulse (red solid line) and the traditional π-pulse (blue dashed line) under detuning error $\delta_{\Delta }$ and laser intensity fluctuation error $\delta_{\Omega }$.

This error is eliminated when α=−1, which gives $\left|\int_{0}^{T}-i2\dot{\theta }\sin^{2}\theta e^{i\eta (t)}dt\right|=0$. Figure 2a displays the resulting Ω(t) and Δ(t) for α=−0.434 (Doppler-robust) and α=−1 (intensity-robust).

The momentum transfer per transition cycle is defined as

(23) Δp=−ℏkP,

where *P* denotes the population inversion probability. Under ideal conditions (P=1), Δp=−ℏk. Figure 2b compares the deceleration performance of STA-optimized pulses versus conventional π-pulses under detuning errors $\delta_{\Delta }$ and intensity fluctuations $\delta_{\Omega }$, demonstrating the superior robustness of the STA protocol.

Applying the STA protocol to Raman cooling, we map the three-level system to an effective two-level model with control parameters derived from Equation (19):

(24) $$\begin{array}{cc} \Omega_{\mathrm{eff}} & =-\dot{\theta }/\sin\beta ,\\ \Delta_{\mathrm{eff}} & =\dot{\theta }\cot\theta \cot\beta -\dot{\beta }. \end{array}$$

For two-photon detuning errors, the inversion probability follows Equation (20), with error cancellation achieved at α=−0.434.

Intensity fluctuations $\Omega_{j}\to \Omega_{j}\left(1+\delta_{\Omega }\right)$ (j=P,S) yield the modified inversion probability

(25) $$P\approx 1-\delta_{\Omega }^{2}{\left|\int_{0}^{T}\left(\Delta_{\mathrm{eff}}\sin\theta -i2\dot{\theta }\sin^{2}\theta \right)e^{i\eta (t)}dt\right|}^{2},$$

where we retain only first-order terms in $\delta_{\Omega }$. The error cancellation occurs at α=0.2502.

**Figure 3:**
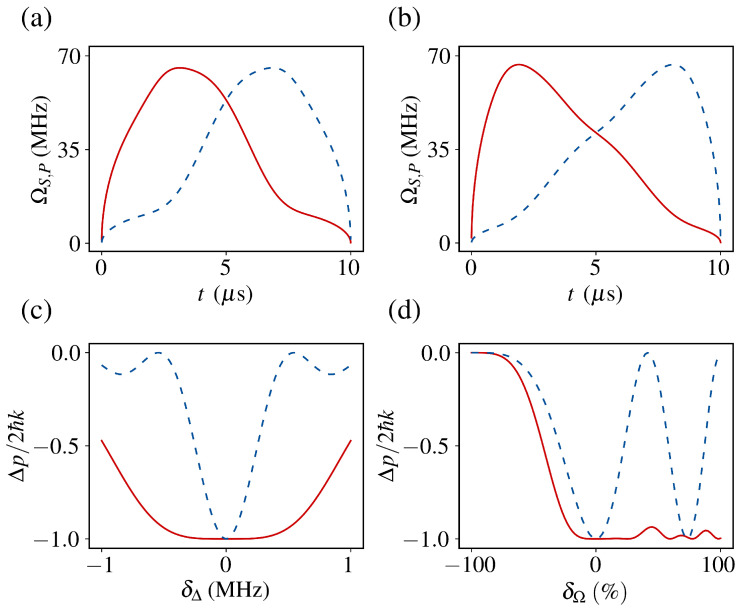
Pulse profiles and deceleration performance for the effective two-level system. (**a**,**b**) Temporal profiles of $\Omega_{S}$ (red) and $\Omega_{P}$ (blue) from the effective two-level model with Δ=1 GHz. (**c**,**d**) Deceleration performance under $\delta_{\Delta }$ and $\delta_{\Omega }$ for STA (red) vs. π-pulse (blue).

Maintaining large detuning (Δ=1 GHz), we compute $\Delta_{\mathrm{eff}}$ and $\Omega_{\mathrm{eff}}$ for both α values using Equation (19), obtaining the corresponding $\Omega_{P}\left(t\right)$ and $\Omega_{S}\left(t\right)$ shown in Figure 3a,b.

The momentum transfer per cycle in the three-level system is

(26) Δp=−2ℏkP,

reaching Δp=−2ℏk under ideal conditions (P=1). Figure 3c compares deceleration performance between STA-optimized and π-pulses under simultaneous detuning errors ($\delta_{\Delta }$) and intensity fluctuations ($\delta_{\Omega }$). The STA protocol demonstrates significantly enhanced robustness, achieving superior cooling precision through reduced sensitivity to experimental imperfections.

### 3.2. Dissipation Analysis

In realistic laser cooling, dissipative effects from spontaneous emission must be accounted for. Spontaneous decay from the excited state introduces momentum diffusion, broadening the atomic momentum distribution. These dissipative dynamics follow the Lindblad master equation

(27) $$\frac{d\rho }{dt}=-\frac{i}{\hbar }\left[H,\rho \right]+\gamma \left(L\rho L^{†}-\frac{1}{2}\left\{L^{†}L,\rho \right\}\right),$$

where γ=1/τ is the spontaneous emission rate (τ: excited-state lifetime) and the jump operator $L=|\tilde{1}\rangle \langle \tilde{2}|$ describes dissipation. For the two-level subsystem, the excited-state population decays at rate γ.

**Figure 4:**
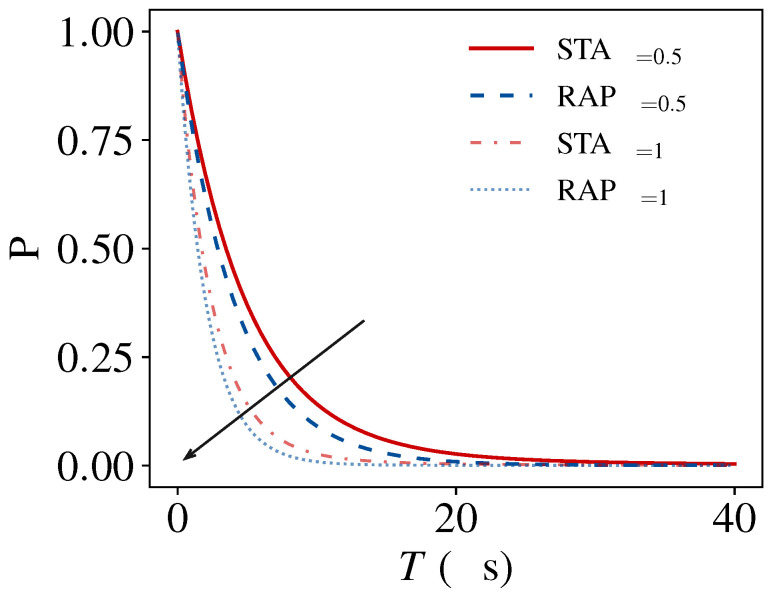
Comparison of momentum transfer efficiencies in dissipative environments. Transfer probability as a function of operation time *T* for the STA pulse (red solid and light red dot-dashed lines) and RAP pulse (blue dashed and light blue dotted lines) under decay rates γ=0.5 and γ=1, respectively. Arrows indicate the direction of increasing γ.

Figure 4 reveals the scaling of the momentum transfer efficiency with operation time *T*. The inversion probability *P* decays approximately as 1/T under dissipation, demonstrating the fidelity–speed trade-off. The STA protocol shows marginally enhanced robustness compared to conventional RAP at equal γ. All schemes exhibit a systematic reduction in efficiency with increasing *T*, confirming the fundamental time-dissipation constraint. Longer operation accumulates decoherence and momentum diffusion from spontaneous emission. By supressing *T* below $\gamma^{-1}$ while maintaining precision, STA provides a way to avoid this limit.

For three-level Raman cooling, large detuning ($\Delta \gg \Omega_{P},\Omega_{S}$) with two-photon resonance suppresses the excited-state population to $O[{\left(\Omega /\Delta \right)}^{2}]\ll 1$. The system then approximates an effective two-level model with analogous dissipation. STA achieves momentum transfer in the limit $T\ll \gamma^{-1}$ via tailored $\Omega_{\mathrm{eff}}\left(t\right)$ and $\Delta_{\mathrm{eff}}\left(t\right)$, minimizing dissipation-induced momentum diffusion. On the contrary, conventional RAP requires $T\propto \Delta /\Omega^{2}$ to satisfy adiabaticity, leading to significant accumulation of dissipation. Thus, STA enhances robustness through time-optimal control fields operating below the dissipative threshold.

## 4. Conclusions and Outlook

Motivated by the inherent limitations of conventional techniques—where adiabatic methods (RAP/STIRAP) require prolonged operation times susceptible to spontaneous emission, while π-pulse schemes lack resilience against Doppler shifts and laser noise—we developed optimized STA control fields through inverse engineering of Lewis–Riesenfeld invariants. This framework integrates time-dependent perturbation theory to derive noise cancellation conditions, paired with polynomial ansatz solutions for the auxiliary angle parameter, thereby yielding analytical pulse profiles for both the detuning Δ(t) and Rabi frequency Ω(t). For three-level Raman systems, this framework is extended by constructing an effective two-level model via large-detuning adiabatic elimination, facilitating the direct implementation of the STA protocol for counter-propagating laser fields. Notably, whereas conventional adiabatic protocols incur substantial momentum diffusion induced by spontaneous emission decay owing to prolonged operation times, STA circumvents this limitation by shortening pulse durations, thereby suppressing losses arising from spontaneous emission. Consequently, our framework exhibits enhanced cooling efficiency in both two-level systems and three-level Raman configurations, achieving >99% momentum transfer efficiency under ±20% laser intensity fluctuations and significantly outperforming π-pulses under equivalent Doppler detuning errors. These results establish the theoretical value of STA in quantum control.

Future research should extend this work to open up new quantum systems by directly incorporating dissipation engineering into the optimization framework. Specifically, the quantum optimal control of STA could enable adaptive pulse designs that actively suppress dissipation-induced momentum diffusion. By training deep reinforcement learning (DRL) agents on Lindbladian dynamics, algorithms could gain enhanced robustness against non-Markovian noise, thereby refining control protocols for real-world quantum devices limited by decoherence. The combined STA-DRL framework could be adapted to other applications requiring resilience against parameter drift, such as quantum sensing (where noise suppression enhances precision), fault-tolerant gate operations (enabling error-adapted pulses), and quantum state transfer (mitigating dissipation losses). To validate this, experiments on platforms with tunable dissipation (e.g., cavity QED or trapped ions) would bridge theory and practice. Iteratively combining analytical invariants with data-driven optimization establishes a versatile framework for developing adaptive quantum protocols in noisy environments. Further work should probe these interdisciplinary connections to advance quantum control theory and accelerate scalable quantum technologies.

## Data Availability

The data presented in this study are available on request from the corresponding author.
